# Unlocking the mechanisms of change in the MAMAACT intervention to reduce ethnic disparity in stillbirth and newborns' health: integration of evaluation findings

**DOI:** 10.3389/frhs.2024.1233069

**Published:** 2024-02-16

**Authors:** Sarah Fredsted Villadsen, Helle Johnsen, Trine Damsted Rasmussen, Claus Thorn Ekstrøm, Janne Sørensen, Elie Azria, Janet Rich-Edwards, Birgitta Essén, Ulla Christensen, Signe Smith Jervelund, Anne-Marie Nybo Andersen

**Affiliations:** ^1^ Section of Social Medicine, Department of Public Health, University of Copenhagen, Copenhagen, Denmark; ^2^ Department of Midwifery and Therapeutic Sciences, University College Copenhagen, Copenhagen, Denmark; ^3^ Section of Epidemiology, Department of Public Health, University of Copenhagen, Copenhagen, Denmark; ^4^ Section of Biostatistics, Department of Public Health, University of Copenhagen, Copenhagen, Denmark; ^5^ Section of Health Services Research, Department of Public Health, University of Copenhagen, Copenhagen, Denmark; ^6^ Obstetrical Perinatal and Pediatric Epidemiology Research Team, INSERM, Paris, France; ^7^ Department of Epidemiology, Harvard T.H. Chan School of Public Health, Boston, MA, United States; ^8^ Department of Women's and Children's Health, Uppsala University, Uppsala, Sweden

**Keywords:** emigrants and immigrants, reproductive health, first 1000 days, health inequalities, complex intervention, program evaluation, antenatal care, cultural competence

## Abstract

Ethnic disparities in stillbirth exist in Europe and suboptimal care due to miscommunication is one contributing cause. The MAMAACT intervention aimed to reduce ethnic disparity in stillbirth and newborns' health through improved management of pregnancy complications. The intervention encompassed training of antenatal care midwives in cultural competencies and intercultural communication combined with health education materials for the expecting parents about symptoms of pregnancy complications. The evaluation consisted of a qualitative in-depth implementation analysis and a process evaluation embedded in a cluster randomized trial including 19 of 20 maternity wards in Denmark. In this article, the findings from the different evaluation perspectives are integrated. The integration follows the principles of realist evaluation by analyzing to what extent the MAMAACT activities were generating mechanisms of change in interaction with the context. The integration analysis shows that the health education materials in the MAMAACT intervention contributed to heightened health literacy concerning pregnancy complications among pregnant women. Additionally, the training of midwives in cultural competency and intercultural communication raised awareness among midwives. Nonetheless, the exclusive emphasis on midwives and the inflexibility in care provision hindered them from changing their communication practices. To enhance the cultural competence in maternity care, it is essential to implement more comprehensive initiatives involving healthcare professionals in maternity care at all levels, from pregraduate to postgraduate. Adequate interpreter services and management support should also be ensured. Currently, the Danish antenatal care system faces challenges including inadequate information transfer between healthcare sectors, insufficient differentiation of care, and inflexibility in midwife scheduling. This results in a lack of responsiveness to the individual needs of women with immigrant backgrounds, potentially reproducing health inequities.

## Introduction

Currently in Europe, significant inequities in stillbirth and infant mortality rates persist with migrant mothers born in Low- and Middle Income Countries (LMIC) and their children facing higher risks than native populations (1, 2). The relationship between migration and pregnancy outcomes unfolds in heterogenous ways according to the country of origin, the reason for migration, and the resettlement county context. The disparities are rooted in a complex interplay of various structural elements, which also encompass the positioning of an individual's attributes, such as skin color, gender and socioeconomic position, within the broader societal context (3). Despite universal access to maternity care in most European countries, suboptimal maternity care is more prevalent in women with immigrant backgrounds compared to women without a migration history; the women with immigrant backgrounds make less use of and receive lower quality maternity care (1, 4). In 2002, a Swedish perinatal audit documented that communication barriers were a leading cause of the higher risk of perinatal death (5).

The term immigrant refers to individuals, who moved away from their usual country of residence (6), while ethnicity is a subjective term, encompassing the social groups to which people feel they belong based on multiple aspects like language, culture, and religion (7). In Denmark, the history of immigration from outside Europe is relatively short, and currently, relatively few women of reproductive age are born in Denmark to parents with immigrant backgrounds. Thus, the terms ethnic minorities and immigrants in Denmark are highly overlapping. In this article, the term ethnic minority refers to women who immigrated to Denmark, and ethnic disparity reflects differences according to immigration background. Immigration to Europe has been increasing, and in 2021, 20% of births in Denmark were to women with immigrant backgrounds (8).

Consequently, healthcare systems need to adapt to serve an increasingly heterogeneous population. To adapt requires that the communication barriers during healthcare encounters are addressed (9, 10), encompassing both linguistic and cultural aspects (11). Health communication and counselling for pregnant women are paramount to prevent poor pregnancy outcomes and ensure a positive pregnancy experience (12). Language barriers (13) and lack of good interpretation services (14), lower health literacy levels (15), and prejudicial attitudes and discrimination from healthcare providers (1) are potential barriers to equitable communication and care. A recent Norwegian study found that more than one-third of women with immigrant backgrounds did not understand the information provided during maternity care and 50% were unaware of whom to contact in case of pregnancy-related complications (16).

Different approaches to address these inequalities and barriers to quality maternity care for women with immigrant backgrounds have been initiated. Evaluations from Sweden and Norway of group antenatal care models and the use of doulas have shown a potential to improve the quality of the communication and interaction with maternity care providers, however, the evidence of positive effects on clinical practice and health outcomes is vague, possibly due to methodological limitations (17, 18). Another approach has been the training of healthcare professionals in cultural competence (10, 19, 20), i.e., the obligation for healthcare providers to accept and respect diversity, consistently engage in introspection regarding the dynamics of such differences and adapt their services to cater to the needs of diverse populations (10). The Oramma project implemented cultural competence training of midwives in Greece, the Netherlands and the United Kingdom and found positive effects on midwives' knowledge and self-perceived cultural competence. Nevertheless, there is still a gap in knowledge regarding how to address disparities in maternity care for women with immigrant backgrounds, specifically in relation to the effectiveness of cultural competence training as a means to enhance clinical practice and improve health outcomes (19, 21, 22).

In Denmark, the MAMAACT intervention from 2012 to 2023 has been the most comprehensive effort in this field. The MAMAACT intervention was designed, feasibility tested, implemented, and evaluated to reduce social and ethnic disparity in stillbirth and newborns' health through improved management of pregnancy complications (23). In short, the intervention consisted of training of antenatal care midwives in cultural competencies and intercultural communication combined with health education materials for the expecting parents about symptoms of pregnancy complications. The principles of proportional universalism (24) were used and the intervention was given to all expecting parents regardless of ethnicity. The midwives were trained to communicate tailored to the individual needs of all women, as the aim was to reduce both social and ethnic disparity and targeting all non-Danish-born women only would not reflect the heterogeneity of non-Danish-born women and potentially induce stigma. The MAMAACT intervention was evaluated in a nationwide cluster-randomized effectiveness trial. In the trial, an in-depth qualitative implementation analysis and a mixed methods process evaluation were embedded. Only women born in LMIC were included in the qualitative implementation analysis, whereas the quantitative effectiveness evaluation reported findings for both the total population and for a subsample of women immigrated from LMIC.

To acknowledge that healthcare systems and human behaviors are complex and dynamic (25), the intervention was designed as a complex intervention (26). Research in complex interventions goes beyond analysing intervention effects as it explores how the intervention works for whom and under what circumstances (27). It can be argued that in public health, no single method or study design is superior in obtaining evidence, but the integration of findings across methods and disciplines will provide the highest level of evidence (28). A realist evaluation perspective can provide a deeper focus on the interplay between activities and contextual circumstances for generating changes. Hindering and facilitating elements of the context will be illuminated and provide important understanding of needed revisions of the intervention (29). However, a consequence of these comprehensive evaluations is that the findings are published in several articles due to their scope exceeding that of a single article (27). Alvarado et al. argue that the integration of findings from population health interventions can be mutually illuminating and produce findings that are greater than the sum of parts (30). The findings from the MAMAACT evaluation have been published in seven different scientific publications (31–37), but in this article, the findings are integrated. In the integration, the principles of the realist evaluation are used (29). The findings of the implementation analysis, the process evaluation, and the effectiveness evaluation are integrated to illuminate how far the intervention activities contributed to change and what role the context played in this. By integration of these findings, heightened clarity on the most pivotal insights is anticipated. This, in turn, will facilitate comprehensive discussion on the applicability of MAMAACT to other contextual circumstances, providing valuable information for decision-makers. This article has a specific focus on understanding the mechanisms of change for women with immigrant backgrounds from LMIC. The aim of this article is twofold:

(1) to integrate the MAMAACT evaluation results to identify how the activities were affected by contextual enablers and barriers to produce mechanisms of change, and
(2) to analyze which adaptions should be made if the mechanisms of the intervention are to unfold in future interventions to improve pregnancy outcomes of women with immigrant backgrounds in Europe.

## Study setting: antenatal care in Denmark

The Danish welfare state provides free coverage of healthcare services to all with permanent residency. In 2018, amendments to the Danish Health Act were introduced stating that interpreter services require co-payment for residents who have been in Denmark for more than three years. Persons with mental or physical diseases can be exempted from the co-payment. Previously, interpretation was without user payments, and after the amendments, the use of interpreter services dropped (38).

Antenatal care is a shared responsibility of general practitioners (GP) at the primary care level and antenatal services affiliated with the hospital maternity wards at the secondary and tertiary care level (in the following referred to as maternity ward level). Most antenatal care at the maternity ward level is provided by midwives at antenatal clinics located closer to residential areas than the hospitals. Women are enrolled into antenatal care at their GP around gestational weeks 6–10, whereafter women without known risk factors or pregnancy complications have another two visits at the GP (around gestational weeks 24 and 35), and five midwifery visits at the antenatal clinic spread around these time points. The national policy for antenatal care states that care at maternity ward level should be differentiated in four levels of care based on the needs of the women (39). Level one targets women with expected uncomplicated pregnancies and is provided by GPs and midwives. Level two targets women with antenatal, birth and postnatal risks, i.e., women with overweight, previous complicated birth or breastfeeding problems, and is provided by maternity care providers. Level three targets women with complicated somatic or mental illnesses and social vulnerabilities, while level four targets women with complex problems relating to substance abuse, severe psychological or psychiatric disorders, or severe social disadvantages. Antenatal care in levels three and four is provided by an interdisciplinary team including midwives, doctors, nurses, psychologists, psychiatrists, and social workers.

In 2012, around 30% of the maternity wards had targeted care for women with immigrant backgrounds. Here the women attend care with a team of midwives, who have a special interest and experience in intercultural communication. According to the midwives at these places, they have better abilities to use interpreters and more flexibility in their daily schedules (40). In the last two decades, the work environment for midwives has been discussed in Denmark, as midwives have one of the highest prevalences of burnout in Denmark (41).

## The MAMAACT intervention—design and evaluation approach

### Intervention design

The motivation to develop an intervention arose from a comprehensive nationwide register-based study that revealed significant ethnic disparities in stillbirth and infant mortality rates in Denmark (42). A supplementing register-based study was conducted to explore the contributing role of consanguinity, as studies from Norway had shown increased prevalence and associated increased rates of stillbirths among immigrants with Pakistani origin (43). The Danish study indicated that consanguinity only played a minor, if any, role in the increased risk of adverse outcomes in women of immigrant background (44). The intervention development continued with a mixed methods needs assessment. It revealed insufficient needs-based communication tailored to the individual levels of health literacy, insufficient use of interpretation services, and unsystematic provision of health information about symptoms of pregnancy complications in the midwifery-based antenatal care (23). Further, a mini-audit showed that there was a delayed response from both women, midwives and obstetricians in the management of pregnancy complications (45), potentially delaying initiation of treatment of complications and contributing to the increased risk of stillbirth and infant death among immigrant groups in Denmark from LMIC. Therefore, it was chosen to focus on improving the communication about signs of pregnancy complications between antenatal care midwives and pregnant women. The intervention was developed in a co-creation process with researchers, clinical midwives and in partnership with Neighborhood Mothers. Neighborhood Mothers is a non-profit organization, which gathers primarily women with immigrant backgrounds who volunteer to help and support vulnerable women in ethnically diverse neighborhoods.

**Figure 1:**
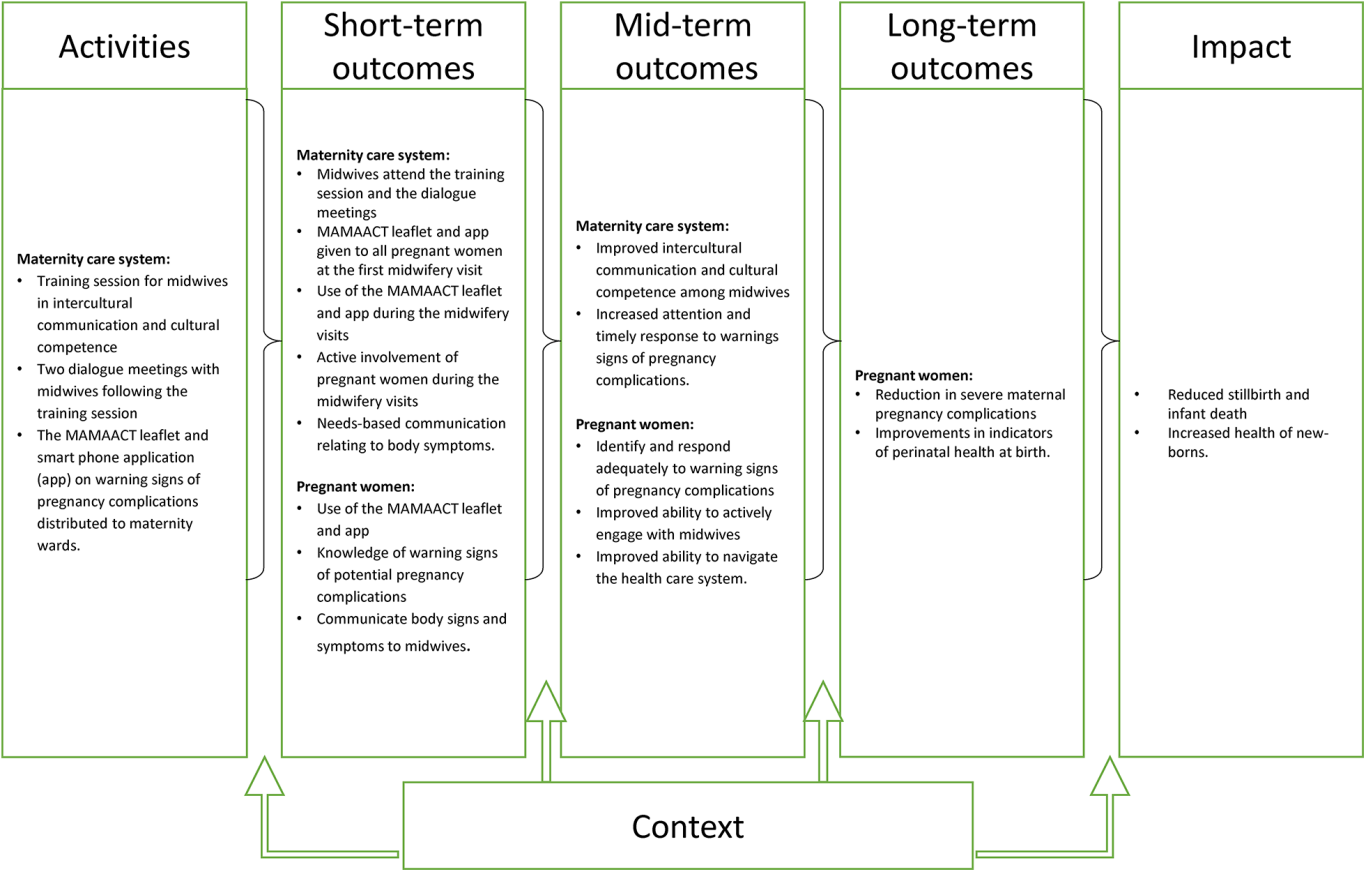
The logic model of the MAMAACT intervention. The figure was published in the article: Damsted Rasmussen et al. (35).

Improved management of complications was expected to be obtained through a two-tiered approach including two main activities: 1) training of midwives in cultural competence and intercultural communication and 2) new health education materials about pregnancy complications. These two activities were together to improve the responsiveness of the midwives to the health literacy level of pregnant women and improve the communication and response to pregnancy complications. Importantly, the intervention was considered to mainly work through the structural level, not putting too much emphasis on the individual responsibility of the women. A logic model was developed to identify and illuminate how the intervention activities were expected to generate outcomes (Figure 1).

The intervention was tested for feasibility at the largest maternity ward in Denmark (31). The feasibility test took place over 10 months in Denmark's largest maternity ward and found that the intervention was acceptable, however needed a few revisions (ibid). The midwives requested more training on how to introduce the leaflet and app to the women, and this was included in the trial version of the training. In the feasibility phase, the intervention also included five extra minutes at the first antenatal care visit for each woman, as the leaflet was to be introduced here. However, due to difficulties with the electronic booking system this was not implemented, and most midwives found it feasible to use the leaflet within the given timeframe. For this reason and considerations of costs and sustainability, the extra five minutes were cancelled in the trial.

In the national trial, the training component for midwives providing antenatal care included a 6-h training day and two smaller group dialogue meetings to continue the reflection and translate learnings into antenatal care practice. The training day was developed using the framework for cultural competence training by Seeleman et al. (46). The concept builds on an understanding that culture is dynamic and a lens through which we understand and perceive ourselves and the needs of others. All human beings categorize and tend to stereotype according to their cultural understanding in encounters with others. Therefore, healthcare providers need knowledge about the diversity of health in immigrant groups and awareness of their own preconceptions. Further, the concept highlights that healthcare providers need abilities to communicate with diverse patients and to be flexible in encounters. Thus, in MAMAACT, the training was designed to make the midwives reflect on their cultural understandings and see the need to move beyond these, to assess the individual women they meet in antenatal care encounters. The ability to communicate beyond own cultural understanding was trained using an intercultural communication component, including the phases in a clinical encounter, active listening, and the need to ask open-ended questions (47, 48).

The training program included the following topics: ethnic differences in stillbirth in Denmark, intercultural communication, case-based communication exercises focusing on needs-based communication, and the use of the MAMAACT information material. The cases were fictive elaborations of perinatal death cases among children of women with an immigrant background from a Danish hospital (45). The case-based learning was facilitated by clinicians from specific specialized migrant medical clinics in Denmark ensuring practice-based compatibility and clinical credibility.

The health information material included a leaflet and an app. Both materials described the most important body symptoms indicating a pregnancy complication and how to contact the healthcare services in each situation. The language was kept in lay terms and used pictograms of the body. It aimed to address nine bodily symptoms that could be warning signs for potentially dangerous conditions such as vaginal bleeding, severe headache and sudden swelling, redness, and heat in one leg. The material was available in the six most frequently spoken languages among pregnant women in Denmark. Priority was given to keep the leaflet brief, whereas the app had a little more explanation about the signs and also included an audio function for women, who preferred listening over reading, and direct dial to the healthcare facilities. To avoid the use of the app in the control group, the download of the app required individual codes found on the leaflet. The midwives were to use the leaflet according to the health literacy levels of the women at the first visit. In subsequent visits, the midwives were trained to refer to the material and to strive for a shared language with the women about bodily symptoms.

### Evaluation approach and data

The intervention was scaled up in a national cluster randomized trial and implemented at 19 out of 20 maternity wards in 2018–2019. Ten wards were randomized to the intervention.

The integration analysis of the current article is based on the following four evaluation components. Firstly, an in-depth qualitative implementation analysis was conducted with participants from five of the ten intervention wards. The data consisted of forty participant observations of midwifery visits, twenty-one in-depth interviews with women originating from LMIC, and nine small focus groups with midwives (an average of three midwives per interview). The aims were to analyze barriers to the mechanisms of change at the organizational antenatal care level (32) and individual level focusing on the everyday life of the women (34). To illuminate the interplay between intervention activities and the context of everyday life of the women, the middle-range theories of situational disease as explained by Alonzo (49) and Gannik et al. (50) were used. Furthermore, we studied how women and midwives' interactional dynamics were affected by the intervention (33). In this article, the middle-range theory of cultural health capital by Shim (51) was applied.

Secondly, a mixed methods process evaluation was conducted to analyze dose, reach, and fidelity (35). The study included data from all 19 trial wards and consisted of standardized information sheets from the dialogue meetings, survey data from pregnant women and app downloads.

Thirdly, a quantitative survey was used to study the intervention effectiveness on women's pregnancy complication-related health literacy. Data were collected from all maternity wards and included 670 pregnant women born in LMIC (37).

Finally, a quantitative evaluation using national registers to analyse indicators of perinatal health in all recent births in Denmark, including 25,400 births from women born in LMIC was conducted (36, 37).

## Integrational analysis of mechanisms and barriers

The integrational analysis follows the two main intervention activities, namely (1) training of midwives for better needs-based communication and (2) health education materials for improved pregnancy complication health literacy. The analysis in each of these dual strands seeks to reveal to what extent the mechanism was activated and why/why not. Subsequently, the integration of these dual strands is examined to ascertain their collaborative effect on health outcomes. The analysis then delves into needed moderations of the intervention, aiming to optimize its functionality within the current context and further enhance its relevance in new contexts.

### Did the MAMAACT training of midwives improve cultural competence?

The process evaluation showed that 87% of the targeted midwives (*n* = 346) attended the training day (35). In the qualitative focus groups, the midwives expressed that the focus on cultural competence and intercultural communication was relevant and important to them. Thus, a readiness to address the ethnic disparity in antenatal care was present. The analysis of the dialogue meetings showed that midwives experienced challenges with engaging women with an immigrant background in communication due to the women being shy, having difficulties in expressing themselves about reproduction and pregnancy, and also having linguistic barriers (35). However, it was also clear that the midwives increased their reflections and curiosity regarding understanding the needs of women with immigrant backgrounds after the training. They expressed to have become aware of the need not to make categorical assumptions about health and behaviors in immigrant groups, but rather ask questions to identify individual needs. Combining these findings illuminate that the attitudes of midwives were positively affected by the intervention. Despite this positive development, it was evident from the participant observations that the midwives did not change their communication style during encounters with pregnant women with immigrant backgrounds towards more active involvement of the women and needs-based dialogue. This was also reflected in the analysis of the individual interviews with the women (33).

In the qualitative implementation analysis, several barriers that hindered the effect of increased awareness and culturally competent attitudes on improved communication practice were documented. The analysis of the organizational antenatal care context showed that the collaboration between GPs and midwives had limitations in providing a seamless transfer of information (32). The GP's assessment of the women's individual needs and requirements to enrolment into differentiated care did not function optimally for many of the women with immigrant backgrounds. Often, the women's previous obstetrical and psychosocial needs were overlooked, and therefore the GPs did not enroll the women into differentiated care beyond level one. The records that were forwarded to midwifery antenatal care often lacked obstetric and psychosocial information why the midwives had to repeat this assessment at the initial encounter. Therefore, the midwife visit was characterized by getting and providing factual information rather than engaging in flexible and needs-based dialogue and this was not improved by the intervention. Information on the women's linguistic abilities in Danish often lacked, and professional interpreters were often not booked even if indicated and needed, consequently reducing the women's ability to communicate and negotiate their needs for services. Reinforcing these communication barriers were the new interpreter amendment, as families would more often refrain from accepting interpreters to avoid the fee. In addition, the midwifery visits were generally characterized by high task loads and restricted time schedules resulting in limited flexibility to adapt the visit to the individual woman's care needs. The midwives did not have the autonomy to refer psychologically vulnerable women directly to psychologists or psychiatrist help (26). All aspects hindering the autonomy and flexibility of midwives.

To explore more in-depth how the intervention affected the communication and interactional dynamics between the midwives and women with immigrant backgrounds, we applied the concept of cultural health capital (51) to the implementation analysis. The concept of cultural health capital illuminates how interactional dynamics can produce unequal treatment. Shim et al. argue that human beings all possess cultural health capital defined as certain skills, attributes, and modes of interaction that are shaped by the social classes we are part of (ibid). Within the healthcare system, a certain cultural health capital is valued higher than others, and without being aware, healthcare providers will communicate easier with patients that possess a cultural health capital that is like their own. Consequently, patients with lower socioeconomic positions will potentially attain less person-centered care compared to patients more similar to the healthcare providers, and these interactional dynamics reproduce health inequalities. The analysis showed that the MAMAACT communication training had limited success in changing the midwife's habitual ways of interacting with pregnant women. Instead, the midwives continued to inform women rather than using a dialogue-based approach, and they neglected to invite the women to participate in setting the agenda for the antenatal visits (33). This dynamic was not only a result of the challenging structures of antenatal care but also the social class-based personal and professional identity forming the habitual ways of midwives engaging with pregnant women.

The effectiveness evaluation informs whether the mechanism of improved needs-based communication was activated even though barriers were documented. In the survey-based effectiveness evaluation, analyses of improvements in women's health literacy regarding their ability to actively engage with healthcare providers were conducted. However, among women with immigrant backgrounds, the mean level of active engagement did not improve. Further, analyzes were conducted using survey data on the women's assessment of whether the antenatal care midwife (1) really listened to what they had to say, and (2) made an effort to get to know issues of individual importance. No effects of the intervention were seen on these items either (52).

Thus, the integration of the qualitative implementation analysis with the process and effectiveness evaluation, revealed that the training of midwives succeeded to raise awareness and improve attitudes towards not categorizing women with immigrant backgrounds. However, the training was not sufficient to change their abilities to generate need-based dialogue and increase the active engagement of women due to contextual barriers and the habitual practice of midwifery. Considering that cultural competence includes both awareness and abilities, the mechanism only came halfway.

### Did the MAMAACT health education materials improve pregnancy complication-related health literacy?

The other important mechanism of the MAMAACT intervention was the health education material to improve the levels of health literacy regarding pregnancy complications among pregnant women. Survey data from the process evaluation (after the intervention in the intervention arm) included answers from 217 women born in LMIC and showed that the leaflet had high reach and both the leaflet and the app were found very useful as the leaflet was distributed to 80% of the women born in LMIC at the first pregnancy visit and, in this group, 62% expressed that it provided them with the information they were able to use during the pregnancy. About a quarter of the women who received the leaflet subsequently downloaded the app, and among them, 73% used the app information during their pregnancy (35).

In the process evaluation and the implementation analysis, the midwives expressed that the material was very relevant to have when communicating with women with low health literacy or with psychosocial vulnerabilities, while the simplicity made it irrelevant for women with high health literacy levels. The midwives appreciated the credibility of the material as an alternative to the women searching for information on the internet (33, 35). Both the midwives and the pregnant women found the availability of several languages very useful, and more languages were requested. In contrast to the intervention logic, the midwives did not use the leaflet or content hereof to foster a shared language about body signs and potential complications at the subsequent visits after distribution at the first visit (33). This was explained by the contextual barriers and the habitual practice of midwifery. Also, the women did not themselves bring questions and reflections about the material into the conversations at subsequent midwifery visits. At home, the women with immigrant backgrounds used the material to learn about the symptoms and some to practice Danish words and health system navigation. Also, women reported using the material to assess their body symptoms. The women had experienced situations where the information material served to assure that their symptoms were not dangerous, and the midwives and the women expressed that the material had also made some women decide to contact the maternity ward (33). Thus, the mechanism was activated.

In the survey-based effectiveness evaluation, it was shown that the women with immigrant backgrounds at intervention maternity wards increased their confidence in how to respond to warning signs of pregnancy complications (37). For the symptoms of “Redness, swelling, and heat in one leg” and “Vaginal bleeding” the women reported increases from 42% to 57% and 77% to 90% respectively. In the control group, similar improvements were shown, thus, the positive effect was not significantly reflected as a difference between the intervention and control group. In the qualitative analysis, we found that some women with immigrant backgrounds had shared pictures of the app on pregnancy groups on Facebook, potentially giving access to the information to women in the control group (ibid). Further, the spread of the intervention to the control group could also be due to midwives in the control group had become more aware of informing the target group about complications as the intervention was not blinded.

In the qualitative implementation analysis, we found that although the women appreciated the MAMAACT leaflet and the app, the women's attention to symptoms was affected by their action spaces in everyday life (34). The concept of situational disease was applied to illuminate how pregnancy complication warning signs were interpreted and assessed in the context of the everyday lives of women. The understanding of containment was included from Alonzo's work (49). Containment entails that if a person can uphold normal roles and engagement in everyday life situations, the symptom will be contained. The concept of action space from Gannik's study (50), was used to analyze how the capacity one has for containing symptoms depends on the structural resources (education, work, family, social network) available for the individual. The analysis showed that the women's responsibilities beyond pregnancy, for example for other children or work, limited their action space and resulted in them not necessarily paying attention to their own body symptoms. Their action space was also affected by their social network and partner. Many had a very limited network in Denmark they could ask for informational and practical help. Moreover, their partners often had non-flexible working conditions and were breadwinners in the family. These circumstances often made the women contain the symptoms and seek acute healthcare for pregnancy-related worries at a late stage, and this was not fully overcome by the new knowledge received from the leaflet or app. Further, the women expressed that previous negative experiences of not being taken seriously or being listened to when presenting symptoms in encounters with maternity care providers could make them refrain from seeking care (34).

By combining the different evaluation findings, we thus conclude that the mechanism of the health education material to improve the levels of health literacy regarding pregnancy complications did succeed to some degree, as the confidence in the management of potential pregnancy complications (symptoms thereof) increased. Nevertheless, the socially disadvantaged circumstances experienced by many women from LMIC posed barriers to their timely access to healthcare, thereby impeding the effectiveness of the MAMAACT intervention despite the knowledge they acquired through it.

### Why or not did the two mechanisms work together to produce changes in clinical outcomes?

The hypothesis behind the intervention was that the training of midwives and the material together would make the midwives provide more needs-based communication about pregnancy complications and that this would make women respond faster to complications, communicate them more clearly to the midwives at acute services, and midwives would ensure management and treatment faster, ultimately improving the health of the newborn and reduce stillbirth. From the integration of evidence from the two mechanisms above, it is clear, that the mechanism of the midwifery training was only effective halfway, but the health education mechanism was producing change, and therefore it was difficult to know if any effect of the intervention on clinical outcomes could be expected.

Nation-covering register-based data were used to study the effect on a composite perinatal mortality and morbidity outcome comparing changes in the outcome from a pre-implementation period (2014–17) to a post-implementation period (2018–19) in the intervention group relative to the control group. The composite outcome included stillbirths, neonatal deaths, Apgar score <7, umbilical arterial pH < 7, admissions to a neonatal intensive care unit (NICU) > 48 h, and NICU admissions for mechanical ventilation (36). The effect on each of these outcomes was also assessed. The intervention had no effect on the composite outcome for women of origin in LMIC. It was indicated that the intervention made the proportion of newborns admitted to NICU increase and the proportion with umbilical arterial pH < 7 decrease (ibid). This could be a potential positive effect of the intervention reflecting that the midwives (who would disseminate and spread their understanding to the obstetricians and neonatologists) had become more alert to the needs of women with immigrant backgrounds and initiation of the delivery of sick and threatened infants occurred faster, leading to more being admitted to NICU, but with lower levels of hypoxia. We have no other clinical insights from the women and their babies regarding the causes of the outcomes, so we are cautious not to overinterpret these findings as results of the intervention. Thus, the register-based study left us with little certainty of effect, most likely due to the outcomes being so distal from the intervention activities and with a wide gap to the survey-based outcomes. With the current knowledge, we conclude that the intervention had no effects on clinical perinatal outcomes.

### Intervention moderations needed

Complex interventions could be understood as events in systems that are adaptive and characterized by emergence and feedback (26). Employing a systems perspective to the MAMAACT intervention entails that the antenatal care system has its own dynamics and midwives work in a web of interactions with other healthcare providers in an organization interacting with other sectors and systems. The complexity of antenatal care and the dependency of the midwives of the antenatal care organization was not sufficiently included in the intervention. It was not anticipated that midwives were unable to adjust their work flexibly when the complex needs of an individual woman were better understood. The following aspects were important intersecting aspects of context that were not part of the intervention but hindered the mechanism. The antenatal care and hospital management levels were not sufficiently involved, which resulted in the demands for more flexibility in care provision not being realized. For example, it was not possible for the midwives to have more time during a visit or to book a new visit out of the normal schedule. The lack of involvement of the obstetric doctors in the intervention likely made the action space for the midwives limited, which was especially important for acute care of pregnancy complications. In the case of complications during pregnancy or birth, the midwives work with reference to an obstetrician, who has the overall responsibility (53). The limited access to and quality of linguistic interpretation services of the general Danish antenatal care system (54) posed a fundamental challenge to needs-based communication, an issue being exacerbated by the recent interpreter amendment to the Danish Health Act (55). Considering the important role of the GPs in Danish antenatal care including the responsibility to assess and differentiate care according to needs (39), it was problematic not to include the GPs in the intervention. Thus, we find it important to highlight that future interventions to improve communication and interactional dynamics with women with immigrant backgrounds, need to consider the wider organizational system around the antenatal care midwives.

A second consideration regarding aspects hindering the mechanisms is whether the training program for midwives was extensive enough to effectively change practices by improving their abilities to communicate in new and more flexible ways. Midwifery education in Denmark is a three-and-a-half-year professional bachelor's degree with extensive clinical training. Thus, midwifery students are trained into the existing fields of social action. The midwives trained in the MAMAACT intervention have had many years of experience in performing their roles as midwives. Introducing the intervention's components of cultural competence and intercultural communication at the midwifery bachelor program simultaneously with the continued education of midwives at maternity wards might have had a greater impact in the longer run. Beyond the extensiveness of the course, the content could have been different, for example focusing more on bias and stereotypes (56). Nevertheless, the challenges of the intervention were related to practices and not reflections. Kleinman and Benson have argued that cultural competence is a process of reflection more than skills (57), and the change of practices might just take more time.

Thirdly, the use of the concept of cultural health capital can help us understand how difficult it can be to change practices related to healthcare encounters. The concept is drawing on Bourdieu's theories including the concept of habitus which imply that we all have durable dispositions that are shaped by the structures we have met over our lifetime and that these dispositions act as cognitive maps that guide our thinking and actions (51). Enrolment in the midwifery program in Denmark requires high academic performance in high school and therefore midwife students predominantly have been growing up in privileged social circumstances. Thus, their perspectives are affected by the privileged classes' dispositions that imprint them to value skills, attributes, and modes of interaction different from those most women with immigrant background possesses. Exploring the encounter and interactional dynamics from the perspective of cultural health capital, we must acknowledge that practices cannot easily be changed, and that the intervention's ambition might have been too naive.

Fourthly, it is interesting that the mechanism of the health education material was more successful than the mechanism of the training of the midwives. The professional understanding of performing the role of a midwife is predominantly rooted in health sciences, where health education and medical information might be more acceptable within this paradigm. However, this is problematic as the communication and interactional barriers in current antenatal care leave some women with immigrant backgrounds with unmet healthcare needs and inequity in outcomes. The health education material might be easier to handle and virtue signaling. At the same time, we stress that the combination of the leaflets and the app, the simplicity and the inbuilt health system navigation made the mechanism of the material relatively strong.

Finally, we should also acknowledge that the capacity of antenatal care and midwives is limited. Women with immigrant backgrounds often experience socioeconomic hardship. Low socioeconomic position is a well-known risk factor for stillbirth and infant death (58). Women with immigrant backgrounds from LMIC giving birth in Denmark in 2005–2016, had significantly lower levels of maternal education and household income (2) compared to Danish-born women. However, the low socioeconomic position only partly explained the increased risk for stillbirth found in these groups compared to Danish-born women (2), which indicates that the causes of the vulnerabilities are intertwined and the intersectionality of ethnic and social inequalities not fully illuminated. The structures of the everyday lives of women in vulnerable positions cannot be changed by the healthcare system alone. Nevertheless, the antenatal care system in a welfare state should be ready to not reproduce inequalities. Enhancing collaboration between antenatal care services and community-level organizations to support the social network and address the needs of women in vulnerable positions, such as Neighborhood mothers, is needed and merits further research.

### Relevance of the MAMAACT intervention in other contexts

The multiple methods evaluation approach guided by the logic model of the MAMAACT intervention enabled gathering of evidence about the mechanisms of change and most important contextual barriers. The use of middle-range theories to understand the interactional dynamics (51) and containment of symptoms (50) added a theoretical level to the conclusions about the mechanisms that enable relevance of the MAMAACT findings to other contexts. Likely, the interactional dynamics between women with immigrant backgrounds and maternity care providers in other countries have similarities to what we have found. How deeply rooted interactional dynamics are should be considered in all similar interventions and the mechanisms of change need to be supported by structural changes in the wider maternity care system. Likewise, the finding that women are likely to delay response to symptoms, if they face a lack of social support from friends, family, and partners, and live with unstable socioeconomic conditions, might also be relevant to other national contexts and should be considered in interventions aiming to improve the response to complications.

The isolated focus on increasing knowledge and awareness of midwives and not affecting the surrounding system could produce an unintended negative mechanism of moral distress among the midwives. Moral distress entails that healthcare professionals are aware of what a correct professional performance would be, but are hindered by external barriers to perform it (59). The midwives' awareness and an improved understanding of the individual needs of women, combined with limited organizational support to pursue the right actions, could cause frustration. This was indicated in the qualitative data. However, at the same time the midwives expressed that they enjoyed focusing on core aspects of their profession and felt that the MAMAACT intervention was doing so. Hence, to avoid frustration it is crucial to enhance the autonomy and flexibility of healthcare providers through a broader systems approach. Previous Danish research indicate that caseload midwifery could be an organization of care that enables better continuity of care and improved relationships between midwives and women and lower the levels of burnout among midwives (60).

The other intervention projects in this field, all used a targeted approach with women with immigrant backgrounds only as their target group and involved healthcare providers, who were especially motivated to address ethnic disparities (18, 22, 61). In the MAMAACT project, all midwives and pregnant women in the intervention wards were included in the intervention. The targeted approaches include a screening of women to assess their fit with the program. For example, the group antenatal care project in Sweden included Somali-born women only. Selection to care based on country of origin entails a categorization that considers all Somali-born women to need extra services. Such screening, selection, and categorization was avoided in the MAMAACT project, however, it might have come with the price of diluting the dose and effect of the intervention among those in most need.

## Discussion

In educational interventions, outcomes directly related to the intervention activities, and outcomes related to simple behavioral changes have been proven easier to document than distal, complex outcomes (62). In the MAMAACT trial, it was difficult to identify the mechanisms of change between increased confidence in how to handle complications and the composite outcome of neonatal morbidity and mortality. It could have been relevant to quantitatively study the cultural competence levels of midwives like in the Oramma project (22), understanding of healthcare information among pregnant women as studied in Norway (16), and the labour and childbirth subscale of the experience of maternity care questionnaire (63), and the migrant-friendly maternity care questionnaire (64). However, most importantly, we lacked good indicators of clinical practice and outcomes. Having had more information about the acute contacts to the maternity wards would have been interesting, but also complex as an increase in contacts could both be positive and negative depending on the cause of the contact being clinically relevant or not. In previous research, the audit approach to systematically assess the clinical practice against guidelines, has been successful to document care inequities for women of immigrant backgrounds (5). Audits represent an interesting (and labor-intensive) approach to the linkage of proximal outcomes to clinical effects and an audit-based assessment of whether the decrease in umbilical arterial pH and increase in NICU admissions were related to improved clinical responsiveness to the symptoms of women in MAMAACT could have been enriching. Previously, it was documented that children born to women with immigrant backgrounds in Sweden were less likely to be admitted to NICU when clinically indicated (low arterial pH and short for gestational age) compared to ethnic majority children (65), suggesting that the potential break of this pattern by the MAMAACT intervention could be an important achievement. Further, as the intervention is affecting maternal complications, it would be interesting to supplement the perinatal outcomes with the mode of delivery and severity of pregnancy complications (preeclampsia being the most precisely measured in the registers). However, the other intervention projects in the field also have challenges to document the effects of the intervention on clinical outcomes. A Swedish project concluded that group antenatal care for women of Somali origin led to more comprehensive care as the voice and narratives of the women were strengthened and the midwives expanded their understanding of the women and their needs (61). An effectiveness evaluation indicated an improved understanding of health information; however, no effect was shown on postpartum depression (17). Further, a Swedish community-based bilingual doulas intervention indicated a potential to lower caesarean section/instrumental delivery, while no significant effect on postpartum depression was shown (18). The interventions all aim to improve the communication and quality of care and all stem from Scandinavian public, welfare maternity care systems, thus having similarities in the mechanisms they aim for and similar contextual conditions, however different intervention activities and outcomes measures. There is a need to learn across the interventions, develop new clinical outcome measures, and have more consistency in the choice of outcomes, which can reflect improved communication and interactional dynamics of importance to maternal and perinatal health and morbidity.

The MAMAACT study is the largest of the mentioned trials in the field (17, 18, 22), involving almost all maternity wards in Denmark, and the only one designed as a cluster-randomised trial. The limitations of the different data sources and specific analytical approaches are in detailed discussed in the original articles. The risk of contamination from intervention to the control group deserves attention, while the role of interdependency within clusters might not be problematic, as the Intra Cluster Class Coefficient was very low in the effectiveness articles (36, 37). The importance of a thorough qualitative implementation analysis and process evaluation within trials could not be stressed enough. Consequently, the need for resources for these large evaluation studies should not be underestimated. The logic model graphically illustrating the programme theory in Figure 1 was used as the backbone for designing the evaluation, however, it does not sufficiently grasp the complexity of the activities, the mechanisms, the outcomes and the modifying effect of context. Elaboration to better understand the role of middle-range theories for mechanisms and elaborations of the context is needed in future work with logic models and evaluations. We find the use of realist principles in the integrational analysis valuable as the level of abstraction facilitates reflections of transferability. Our identification of needed intervention moderation is aligned with realist thinking as the aim of realist evaluation is to provide knowledge that can refine program theories, a process that never ends, as the contextual circumstances constantly change. Such revisions could lead to new program theories that would better grasp how the intervention can lead to the intended outcomes in future iterations or when transferring to new contexts.

## Conclusion

The health education material of the MAMAACT intervention increased health literacy regarding pregnancy complications. The MAMAACT training improved midwives' cultural competency and intercultural communication awareness. However, the isolated focus of midwives and the lack of flexibility in care provision prevented midwives from adjusting their communication practice. To further strengthen the cultural competence of maternity care providers, more comprehensive efforts should also include GPs, midwives, obstetricians, from the pregraduate to the postgraduate levels as well as maternity care and healthcare system leaders, and the interpreter services. Currently, the poor information transfer between healthcare sectors, the insufficient differentiation of care, and the midwives' lack of flexibility in scheduling make the Danish antenatal care system unable to fully meet the individual needs of women with immigrant backgrounds and overcome the reproduction of health inequities.

## Data Availability

The raw data used are sensitive personal data (holding information about country of birth), and therefore cannot be shared. Further inquiries can be directed to the corresponding author.

## Key messages for decision-makers and managers

• Antenatal care record transfer from General Practitioners to midwives should include interpreter needs and social and obstetric history.
• Differentiation of antenatal care should consider the health literacy levels and psychosocial needs of the women.
• Restore funding for free interpretation services, improve the quality of interpretation services, book more time for visits that will require an interpreter.
• Give midwives increased flexibility to adjust the visits duration and scheduling to the individual needs and authority to refer to psychologists/psychiatrists.
• Continue to improve the cultural competence of maternity care providers, including General Practitioners, midwives, and obstetricians, from the pregraduate to the postgraduate levels.
• Involve maternity care and hospital managers in the need to adapt to increased population diversity.
• Maintain the distribution of the MAMAACT health education materials.
• Strengthen social support for women in the community, for example through increased collaboration between maternity care and organizations like Neighborhood Mothers.
